# An unusual diagnosis of hemorrhage during third trimester

**DOI:** 10.11604/pamj.2020.36.98.22790

**Published:** 2020-06-16

**Authors:** Mounir Moukit, Jaouad Kouach

**Affiliations:** 1 Department of Obstetrics and Gynecology, Military Training Hospital Mohammed V, Rabat, Morocco

**Keywords:** Hemorrhage, angiofibroma, pregnancy

## Image in medicine

A 33-year-old multiparous woman, presented for the first time in our department, at 39 weeks gestation, with the chief complaint of spontaneous vaginal bleeding appeared 6 hours before. There was no abdominal pain, uterine contractions and the bleeding was moderate in amount. She had only a third-trimester ultrasound with normal placental insertion site. On general examination, she was afebrile with normal vital signs. Obstetrical examination revealed fundal height corresponding to gestational age without uterine tenderness or rigidity. Fetal heart rate was normal. Vaginal examination revealed a well-circumscribed vascular mass arising from the posterior vaginal wall. A differential diagnosis of benign vaginal tumours (leiomyoma, angiomyofibroblastoma and cellular angiofibroma) or aggressive vaginal tumours was made. Under spinal anesthesia, caesarean section was performed followed (using lithotomy position) by a circular incision around the lesion with clear margins forcing enucleation of the mass; the wound was repaired with No. 0 monocryl sutures. The histopathologic report was vaginal angiofibroma. The postoperative course was uneventful and no recurrence was noted during a follow-up period of 3 years. Despite its rarity, vaginal angiofibroma should be considered in the differential diagnosis of antepartum haemorrhage.

**Figure 1:**
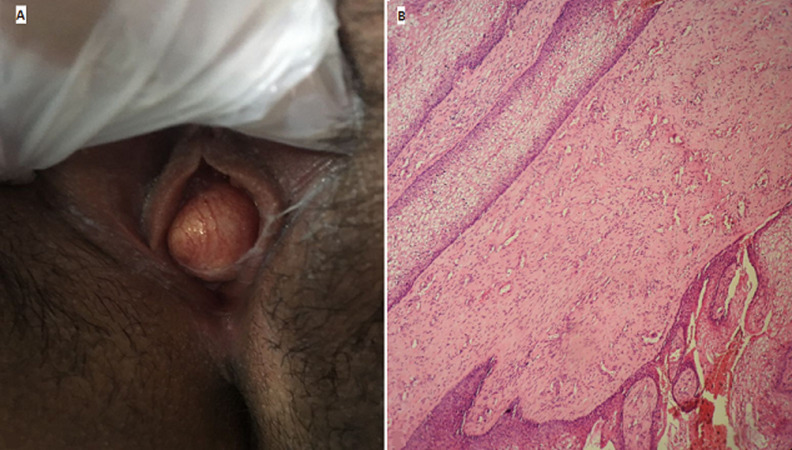
A) vulvoperineal examination objectified the vaginal mass measuring 3cm x 4cm with vaginal discharge; B) spindle and stellate shaped tumour cells with many prominent blood vessels in a fibrous stroma (hematoxylin and eosin x 100)

